# Supplementary material for: Disparity in school children’s reading skills in 11 African countries
Source: PLoS One. 2025 Apr 8;20(4):e0320688. doi: 10.1371/journal.pone.0320688 (PMC11978002; doi:10.1371/journal.pone.0320688)
Supplement: S1 File — S2 Table. Sensitivity test. S2.1 IPW least squares regressions by micro-level factors (outcome variable cutoff at 80%). S2.2 IPW least squares regressions by micro-level factors (outcome variable cutoff at 90%). S2.3 IPW least squares regressions by micro-level factors (continuous outcome variable). S2.4 IPW least squares regressions, interaction terms between various factors and country groups (outcome variable cutoff at 85%, 80%, and 90%). S2.5 IPW least squares regressions, interaction terms between various factors and country groups (continuous outcome variable). S2.6 IPW least squares regressions, interaction terms between disability status and social factors (outcome variable cutoff at 85%, 80%, and 90%). S2.7 IPW least squares regressions, interaction terms between disability status and social factors (continuous outcome variable). (DOCX) [file pone.0320688.s001.docx]

**Supporting information**

**S1 Table. Regression results from first stage of selection model for each country**

| Variable | Central Africa R. | Chad | DRCongo | Ghana | Lesotho | Madagascar | Malawi | The Gambia | Togo | Tunisia | Zimbabwe |
| --- | --- | --- | --- | --- | --- | --- | --- | --- | --- | --- | --- |
| **Disabled** | -0.292* | -0.07 | -0.592*** | -0.440*** | -0.389* | -0.16 | -0.309*** | -0.714** | 0.045 | -0.305 | 0.078 |
| **Location (base category: urban)** | | | |  |  |  |  |  |  |  |  |
|  | -0.280** | -0.253** | -0.197** | -0.290*** | -0.169 | 0.038 | -0.130* | -0.212 | -0.24 | 0.189 | 0.378 |
| **Wealth index (base category=Poorest)** | | | |  |  |  |  |  |  |  |  |
| Second quintile | 0.12 | 0.239* | 0.033 | 0.014 | 0.251* | 0.268** | 0.156** | -0.184 | 0.09 | -0.166 | 0.155 |
| Middle | 0.102 | 0.354** | 0.253*** | 0.208 | 0.353** | 0.367*** | 0.272*** | -0.231 | 0.068 | 0.169 | 0.167 |
| Fourth quintile | 0.300* | 0.323** | 0.490*** | 0.304* | 0.282* | 0.455*** | 0.383*** | -0.28 | 0.117 | 0.288 | 0.747** |
| Richest | 0.343* | 0.507*** | 0.811*** | 0.256 | 0.573*** | 0.334** | 0.539*** | 0.096 | 0.14 | 0.233 | 0.664* |
| **Highest Educational level in the household (base category=No school)** | | | | | |  |  |  |  |  |  |
| Primary | -0.08 | 0.007 | -0.055 | -0.104 | 0.227* | 0.105 | 0.155** | 0.148 | 0.061 | -0.109 | 0.244 |
| Junior High | 0.066 | 0.138 | 0.066 | 0.023 | 0.074 | 0.11 | 0.366*** | 0.159 | -0.094 | -0.128 | 0.28 |
| Senior High+ | 0.047 | 0.085 | 0.066 | 0.185 | -0.148 | 0.047 | 0.568*** | -0.038 | -0.001 | -0.119 | 0.795 |
| **Age (Base category=10)** | |  |  |  |  |  |  |  |  |  |  |
| 11 | -0.018 | 0.021 | 0.053 | 0.098 | 0.202 | 0.12 | 0.196*** | 0.172 | 0.137 | -0.175 | 0.111 |
| e12 | -0.057 | 0.094 | 0.166* | 0.273** | 0.134 | 0.125 | 0.303*** | 0.254 | -0.086 | -0.045 | 0.085 |
| age13 | 0.056 | 0.077 | 0.323*** | 0.349*** | 0.157 | 0.261** | 0.430*** | 0.492** | 0.034 | -0.167 | 0.117 |
| age14 | 0.116 | 0.203 | 0.495*** | 0.631*** | 0.176 | 0.336*** | 0.571*** | 0.512*** | 0.241 | -0.223 | 0.222 |
| **Gender (Base category: Boys)** | -0.167* | 0.063 | -0.091 | -0.099 | 0.290*** | 0.058 | 0.258*** | 0.222* | -0.066 | -0.019 | 0.371*** |
| Constant | 1.133*** | 0.790*** | 0.889*** | 1.704*** | 0.458 | 0.29 | -0.131 | 1.068** | 1.934*** | 1.681*** | -0.08 |
| Sample size | 1458 | 1910 | 3468 | 3159 | 1823 | 2972 | 6332 | 1355 | 1663 | 1669 | 2144 |

**S2 Table. Sensitivity test**

Sensitivity test to the selection of different cutoff thresholds for the outcome variable of reading proficiency. Regression results for the first hypothesis with cutoff points at 80% and 90% are presented in S2.1 Table and S2.2 Table. Regression results for the second hypothesis with cutoff points at 80%, 85%, and 90% are presented in S2.3 Table. Regression results for the third hypothesis with cutoff points at 80%, 85%, and 90% are presented in S2.3 Table. No large sensitivity to the selection of different cutoff thresholds is detected.

S2.1 IPW least squares regressions by micro-level factors (outcome variable cutoff at 80%)

|  | Model1 | Model2 | Model3 | Model4 | Model5 |
| --- | --- | --- | --- | --- | --- |
| **Wealth index (base category=Poorest)** |  |  |  |  |  |
| Second quintile | 0.057*** |  |  |  | 0.042*** |
|  | (0.010) |  |  |  | (0.010) |
| Middle | 0.112*** |  |  |  | 0.080*** |
|  | (0.009) |  |  |  | (0.010) |
| Fourth quintile | 0.208*** |  |  |  | 0.146*** |
|  | (0.010) |  |  |  | (0.011) |
| Richest | 0.372*** |  |  |  | 0.265*** |
|  | (0.010) |  |  |  | (0.012) |
| **Highest Educational level in the household (base category=No school)** | | |  |  |  |
| Primary |  | 0.058*** |  |  | 0.031*** |
|  |  | (0.009) |  |  | (0.009) |
| Junior secondary |  | 0.208*** |  |  | 0.094*** |
|  |  | (0.010) |  |  | (0.010) |
| Senior secondary or higher |  | 0.211*** |  |  | 0.082*** |
|  |  | (0.011) |  |  | (0.011) |
| **Location (base category: urban)** |  |  | -0.225*** |  | -0.087*** |
|  |  |  | (0.008) |  | (0.009) |
| **Disability status (base category: non-disabled)** |  |  |  |  |  |
| Vision disability |  |  |  | 0.032 | 0.022 |
|  |  |  |  | (0.036) | (0.036) |
| Hearing disability |  |  |  | -0.137** | -0.096* |
|  |  |  |  | (0.050) | (0.047) |
| Physical disability |  |  |  | 0.028 | 0.064 |
|  |  |  |  | (0.035) | (0.035) |
| Intellectual disability |  |  |  | -0.165*** | -0.158*** |
|  |  |  |  | (0.016) | (0.016) |
| Multiple disabilities |  |  |  | -0.167*** | -0.119* |
|  |  |  |  | (0.050) | (0.050) |
| **Gender (Base category: Men)** | 0.037*** | 0.042*** | 0.040*** | 0.043*** | 0.035*** |
|  | (0.006) | (0.006) | (0.006) | (0.006) | (0.006) |
| **Age (Base category=10)** |  |  |  |  |  |
| age11 | 0.064*** | 0.067*** | 0.065*** | 0.073*** | 0.063*** |
|  | (0.009) | (0.009) | (0.009) | (0.009) | (0.009) |
| age12 | 0.117*** | 0.121*** | 0.116*** | 0.120*** | 0.115*** |
|  | (0.009) | (0.009) | (0.009) | (0.009) | (0.009) |
| age13 | 0.170*** | 0.176*** | 0.171*** | 0.175*** | 0.170*** |
|  | (0.009) | (0.009) | (0.009) | (0.009) | (0.009) |
| age14 | 0.216*** | 0.227*** | 0.222*** | 0.229*** | 0.215*** |
|  | (0.009) | (0.009) | (0.009) | (0.009) | (0.009) |
| **Country (Base category=Central Africa R.)** |  |  |  |  |  |
| Chad | 0.039* | 0.077*** | 0.082*** | 0.033 | 0.065*** |
|  | (0.016) | (0.018) | (0.018) | (0.018) | (0.017) |
| DRCongo | 0.092*** | -0.021 | 0.045** | 0.002 | 0.064*** |
|  | (0.014) | (0.016) | (0.015) | (0.016) | (0.015) |
| Ghana | 0.348*** | 0.292*** | 0.310*** | 0.300*** | 0.338*** |
|  | (0.015) | (0.017) | (0.016) | (0.019) | (0.015) |
| Lesotho | 0.490*** | 0.439*** | 0.470*** | 0.409*** | 0.492*** |
|  | (0.017) | (0.017) | (0.018) | (0.018) | (0.017) |
| Madagascar | 0.416*** | 0.400*** | 0.424*** | 0.373*** | 0.429*** |
|  | (0.016) | (0.017) | (0.017) | (0.018) | (0.016) |
| Malawi | 0.378*** | 0.370*** | 0.426*** | 0.334*** | 0.407*** |
|  | (0.014) | (0.015) | (0.015) | (0.015) | (0.015) |
| The Gambia | 0.239*** | 0.227*** | 0.173*** | 0.164*** | 0.238*** |
|  | (0.018) | (0.019) | (0.019) | (0.020) | (0.018) |
| Togo | 0.276*** | 0.243*** | 0.259*** | 0.216*** | 0.284*** |
|  | (0.018) | (0.019) | (0.019) | (0.020) | (0.017) |
| Tunisia | 0.772*** | 0.716*** | 0.686*** | 0.716*** | 0.736*** |
|  | (0.013) | (0.014) | (0.015) | (0.015) | (0.014) |
| Zimbabwe | 0.440*** | 0.369*** | 0.440*** | 0.385*** | 0.431*** |
|  | (0.015) | (0.017) | (0.016) | (0.018) | (0.015) |
| Constant | -0.177*** | -0.107*** | 0.123*** | 0.017 | -0.116*** |
|  | (0.016) | (0.016) | (0.016) | (0.015) | (0.019) |
| Sample size | 23591 | 23572 | 23591 | 23591 | 23572 |
| R2 | 0.225 | 0.186 | 0.199 | 0.163 | 0.237 |

S2.2 IPW least squares regressions by micro-level factors (outcome variable cutoff at 90%)

|  | Model1 | Model2 | Model3 | Model4 | Model5 |
| --- | --- | --- | --- | --- | --- |
| **Wealth index (base category=Poorest)** |  |  |  |  |  |
| Second quintile | 0.055*** |  |  |  | 0.042*** |
|  | (0.009) |  |  |  | (0.009) |
| Middle | 0.101*** |  |  |  | 0.073*** |
|  | (0.009) |  |  |  | (0.009) |
| Fourth quintile | 0.186*** |  |  |  | 0.131*** |
|  | (0.009) |  |  |  | (0.010) |
| Richest | 0.337*** |  |  |  | 0.240*** |
|  | (0.010) |  |  |  | (0.012) |
| **Highest Educational level in the household (base category=No school)** | | |  |  |  |
| Primary |  | 0.046*** |  |  | 0.022** |
|  |  | (0.008) |  |  | (0.008) |
| Junior secondary |  | 0.185*** |  |  | 0.083*** |
|  |  | (0.009) |  |  | (0.009) |
| Senior secondary or higher |  | 0.190*** |  |  | 0.075*** |
|  |  | (0.011) |  |  | (0.011) |
| **Location (base category: urban)** |  |  | -0.203*** |  | -0.078*** |
|  |  |  | (0.008) |  | (0.009) |
| **Disability status (base category: non-disabled)** |  |  |  |  |  |
| Vision disability |  |  |  | 0.022 | 0.013 |
|  |  |  |  | (0.036) | (0.036) |
| Hearing disability |  |  |  | -0.127** | -0.090* |
|  |  |  |  | (0.047) | (0.045) |
| Physical disability |  |  |  | 0.038 | 0.071 |
|  |  |  |  | (0.036) | (0.036) |
| Intellectual disability |  |  |  | -0.148*** | -0.141*** |
|  |  |  |  | (0.015) | (0.014) |
| Multiple disabilities |  |  |  | -0.142** | -0.099* |
|  |  |  |  | (0.048) | (0.048) |
| **Gender (Base category: Men)** | 0.034*** | 0.038*** | 0.037*** | 0.040*** | 0.032*** |
|  | (0.006) | (0.006) | (0.006) | (0.006) | (0.006) |
| **Age (Base category=10)** |  |  |  |  |  |
| age11 | 0.054*** | 0.057*** | 0.055*** | 0.063*** | 0.053*** |
|  | (0.009) | (0.009) | (0.009) | (0.009) | (0.009) |
| age12 | 0.093*** | 0.096*** | 0.092*** | 0.096*** | 0.091*** |
|  | (0.008) | (0.009) | (0.009) | (0.009) | (0.008) |
| age13 | 0.135*** | 0.139*** | 0.135*** | 0.140*** | 0.135*** |
|  | (0.009) | (0.009) | (0.009) | (0.009) | (0.009) |
| age14 | 0.173*** | 0.183*** | 0.178*** | 0.185*** | 0.172*** |
|  | (0.009) | (0.009) | (0.009) | (0.010) | (0.009) |
| **Country (Base category=Central Africa R.)** |  |  |  |  |  |
| Chad | 0.040** | 0.074*** | 0.079*** | 0.035* | 0.062*** |
|  | (0.015) | (0.016) | (0.016) | (0.016) | (0.015) |
| DRCongo | 0.073*** | -0.030* | 0.031* | -0.008 | 0.046*** |
|  | (0.013) | (0.014) | (0.013) | (0.014) | (0.013) |
| Ghana | 0.283*** | 0.232*** | 0.248*** | 0.239*** | 0.273*** |
|  | (0.014) | (0.016) | (0.015) | (0.017) | (0.014) |
| Lesotho | 0.361*** | 0.316*** | 0.342*** | 0.288*** | 0.363*** |
|  | (0.017) | (0.018) | (0.018) | (0.019) | (0.017) |
| Madagascar | 0.290*** | 0.277*** | 0.298*** | 0.252*** | 0.303*** |
|  | (0.015) | (0.016) | (0.016) | (0.017) | (0.015) |
| Malawi | 0.258*** | 0.251*** | 0.301*** | 0.218*** | 0.284*** |
|  | (0.013) | (0.013) | (0.014) | (0.014) | (0.014) |
| The Gambia | 0.196*** | 0.183*** | 0.137*** | 0.128*** | 0.193*** |
|  | (0.016) | (0.017) | (0.017) | (0.018) | (0.016) |
| Togo | 0.204*** | 0.174*** | 0.189*** | 0.150*** | 0.211*** |
|  | (0.015) | (0.016) | (0.016) | (0.017) | (0.015) |
| Tunisia | 0.680*** | 0.630*** | 0.602*** | 0.629*** | 0.647*** |
|  | (0.014) | (0.015) | (0.015) | (0.015) | (0.015) |
| Zimbabwe | 0.428*** | 0.365*** | 0.428*** | 0.379*** | 0.420*** |
|  | (0.015) | (0.017) | (0.016) | (0.017) | (0.015) |
| Constant | -0.178*** | -0.111*** | 0.092*** | -0.003 | -0.121*** |
|  | (0.015) | (0.015) | (0.015) | (0.014) | (0.019) |
| Sample size | 23591 | 23572 | 23591 | 23591 | 23572 |
| R2 | 0.182 | 0.148 | 0.16 | 0.128 | 0.193 |

S2.3 IPW least squares regressions by micro-level factors (continuous outcome variable)

|  | Model1 | Model2 | Model3 | Model4 | Model5 |
| --- | --- | --- | --- | --- | --- |
| **Wealth index (base category=Poorest)** | |  |  |  |  |
| Second quintile | 0.064*** |  |  |  | 0.051*** |
|  | (0.008) |  |  |  | (0.008) |
| Middle | 0.117*** |  |  |  | 0.089*** |
|  | (0.008) |  |  |  | (0.008) |
| Fourth quintile | 0.197*** |  |  |  | 0.143*** |
|  | (0.008) |  |  |  | (0.009) |
| Richest | 0.330*** |  |  |  | 0.237*** |
|  | (0.008) |  |  |  | (0.010) |
| **Highest Educational level in the household (base category=No school)** | | | | |  |
| Primary |  | 0.054*** |  |  | 0.029*** |
|  |  | (0.008) |  |  | (0.007) |
| Junior secondary |  | 0.185*** |  |  | 0.086*** |
|  |  | (0.008) |  |  | (0.008) |
| Senior secondary or higher |  | 0.195*** |  |  | 0.083*** |
|  |  | (0.009) |  |  | (0.009) |
| **Location (base category: urban)** |  |  | -0.193*** | | -0.071*** |
|  |  |  | 0.006 |  | 0.007 |
| **Disability status (base category: non-disabled)** | |  |  |  |  |
| Vision disability |  |  |  | 0.025 | 0.017 |
|  |  |  |  | (0.026) | (0.025) |
| Hearing disability |  |  |  | 0.026 | 0.025 |
|  |  |  |  | (0.047) | (0.045) |
| Physical disability |  |  |  | 0.000 | 0.031 |
|  |  |  |  | (0.028) | (0.028) |
| Intellectual disability |  |  |  | -0.170*** | -0.164*** |
|  |  |  |  | (0.014) | (0.013) |
| Multiple disabilities |  |  |  | -0.164*** | -0.123** |
|  |  |  |  | (0.044) | (0.043) |
| **Gender (Base category: Men)** | 0.037*** | 0.042*** | 0.040*** | 0.043*** | 0.036*** |
|  | (0.005) | (0.005) | (0.005) | (0.005) | (0.005) |
| **Age (Base category=10)** |  |  |  |  |  |
| age11 | 0.070*** | 0.073*** | 0.070*** | 0.078*** | 0.069*** |
|  | (0.007) | (0.008) | (0.008) | (0.008) | (0.007) |
| age12 | 0.120*** | 0.124*** | 0.120*** | 0.123*** | 0.119*** |
|  | (0.007) | (0.007) | (0.007) | (0.008) | (0.007) |
| age13 | 0.173*** | 0.178*** | 0.173*** | 0.177*** | 0.173*** |
|  | (0.007) | (0.007) | (0.007) | (0.007) | (0.007) |
| age14 | 0.218*** | 0.227*** | 0.223*** | 0.229*** | 0.217*** |
|  | (0.007) | (0.007) | (0.007) | (0.007) | (0.007) |
| **Country (Base category=Central Africa R.)** | |  |  |  |  |
| Chad | 0.062*** | 0.096*** | 0.098*** | 0.055** | 0.083*** |
|  | (0.016) | (0.018) | (0.017) | (0.018) | (0.016) |
| DRCongo | 0.109*** | 0.009 | 0.068*** | 0.03 | 0.080*** |
|  | (0.014) | (0.016) | (0.015) | (0.017) | (0.014) |
| Ghana | 0.374*** | 0.325*** | 0.339*** | 0.331*** | 0.365*** |
|  | (0.014) | (0.016) | (0.015) | (0.018) | (0.013) |
| Lesotho | 0.503*** | 0.458*** | 0.484*** | 0.430*** | 0.503*** |
|  | (0.015) | (0.016) | (0.016) | (0.017) | (0.015) |
| Madagascar | 0.463*** | 0.450*** | 0.470*** | 0.426*** | 0.474*** |
|  | (0.014) | (0.015) | (0.015) | (0.016) | (0.013) |
| Malawi | 0.409*** | 0.402*** | 0.449*** | 0.370*** | 0.431*** |
|  | (0.013) | (0.014) | (0.014) | (0.015) | (0.013) |
| The Gambia | 0.278*** | 0.267*** | 0.219*** | 0.209*** | 0.277*** |
|  | (0.016) | (0.018) | (0.017) | (0.019) | (0.016) |
| Togo | 0.276*** | 0.248*** | 0.260*** | 0.223*** | 0.283*** |
|  | (0.017) | (0.018) | (0.018) | (0.019) | (0.016) |
| Tunisia | 0.687*** | 0.637*** | 0.612*** | 0.636*** | 0.654*** |
|  | (0.012) | (0.013) | (0.014) | (0.014) | (0.013) |
| Zimbabwe | 0.491*** | 0.429*** | 0.490*** | 0.442*** | 0.481*** |
|  | (0.013) | (0.015) | (0.014) | (0.016) | (0.013) |
| Constant | -0.062*** | 0.005 | 0.208*** | 0.121*** | -0.014 |
|  | (0.014) | (0.015) | (0.015) | (0.015) | (0.017) |
| Sample size | 23591 | 23572 | 23591 | 23591 | 23572 |
| R2 | 0.299 | 0.256 | 0.268 | 0.229 | 0.316 |

S2.4 IPW least squares regressions, interaction terms between various factors and country groups (outcome variable cutoff at 85%, 80%, and 90%)

|  | Family Schooling | | | | | Poverty Status | | | | | | Urban Vs. Rural | | | | | | Disability Status | | | | | |  |
| --- | --- | --- | --- | --- | --- | --- | --- | --- | --- | --- | --- | --- | --- | --- | --- | --- | --- | --- | --- | --- | --- | --- | --- | --- |
| Cut point | 0.85 | 0.8 | | 0.9 | | 0.85 | | 0.8 | | 0.9 | | 0.85 | | 0.8 | | 0.9 | | 0.85 | | 0.8 | | 0.9 | |  |
| **Highest educational level in the household (base category=No school)** | | |  | |  | |  | |  | |  | |  | |  | |  | |  | |  | |  | |
| Primary |  |  | |  | | 0.034*** | | 0.036*** | | 0.018* | | 0.028*** | | 0.029*** | | 0.013 | | 0.026** | | 0.028** | | 0.01 | |  |
|  |  |  | |  | | (0.008) | | (0.009) | | (0.008) | | (0.008) | | (0.008) | | (0.008) | | (0.008) | | (0.008) | | (0.008) | |  |
| Junior secondary |  |  | |  | | 0.100*** | | 0.095*** | | 0.091*** | | 0.069*** | | 0.062*** | | 0.062*** | | 0.067*** | | 0.060*** | | 0.060*** | |  |
|  |  |  | |  | | (0.010) | | (0.010) | | (0.009) | | (0.010) | | (0.010) | | (0.009) | | (0.010) | | (0.010) | | (0.009) | |  |
| Senior secondary or higher |  |  | |  | | 0.116*** | | 0.118*** | | 0.103*** | | 0.080*** | | 0.081*** | | 0.069*** | | 0.081*** | | 0.082*** | | 0.071*** | |  |
|  |  |  | |  | | (0.010) | | (0.010) | | (0.010) | | (0.010) | | (0.010) | | (0.010) | | (0.010) | | (0.010) | | (0.010) | |  |
| No School | 0.054*** | 0.056*** | | 0.049*** | |  | |  | |  | |  | |  | |  | |  | |  | |  | |  |
|  | (0.011) | (0.011) | | (0.010) | |  | |  | |  | |  | |  | |  | |  | |  | |  | |  |
| No School#Mid-reading country | -0.163*** | -0.169*** | | -0.125*** | |  | |  | |  | |  | |  | |  | |  | |  | |  | |  |
|  | (0.015) | (0.016) | | (0.014) | |  | |  | |  | |  | |  | |  | |  | |  | |  | |  |
| No School#High-reading country | -0.102*** | -0.088*** | | -0.129*** | |  | |  | |  | |  | |  | |  | |  | |  | |  | |  |
|  | (0.024) | (0.024) | | (0.024) | |  | |  | |  | |  | |  | |  | |  | |  | |  | |  |
| **Wealth index (base category=Poorest)** |  |  | |  | |  | |  | |  | |  | |  | |  | |  | |  | |  | |  |
| Second quintile | 0.043*** | 0.042*** | | 0.042*** | |  | |  | |  | | 0.040*** | | 0.039*** | | 0.037*** | | 0.043*** | | 0.042*** | | 0.041*** | |  |
|  | (0.010) | (0.010) | | (0.009) | |  | |  | |  | | (0.010) | | (0.010) | | (0.009) | | (0.010) | | (0.010) | | (0.009) | |  |
| Middle | 0.074*** | 0.079*** | | 0.072*** | |  | |  | |  | | 0.070*** | | 0.075*** | | 0.065*** | | 0.074*** | | 0.079*** | | 0.071*** | |  |
|  | (0.010) | (0.010) | | (0.009) | |  | |  | |  | | (0.010) | | (0.010) | | (0.009) | | (0.010) | | (0.010) | | (0.009) | |  |
| Fourth quintile | 0.142*** | 0.144*** | | 0.128*** | |  | |  | |  | | 0.137*** | | 0.139*** | | 0.120*** | | 0.140*** | | 0.143*** | | 0.124*** | |  |
|  | (0.011) | (0.011) | | (0.010) | |  | |  | |  | | (0.011) | | (0.011) | | (0.010) | | (0.011) | | (0.011) | | (0.010) | |  |
| Richest | 0.253*** | 0.261*** | | 0.238*** | |  | |  | |  | | 0.245*** | | 0.254*** | | 0.225*** | | 0.247*** | | 0.256*** | | 0.227*** | |  |
|  | (0.012) | (0.012) | | (0.012) | |  | |  | |  | | (0.012) | | (0.012) | | (0.012) | | (0.012) | | (0.012) | | (0.012) | |  |
| Poor |  |  | |  | | -0.043*** | | -0.046*** | | -0.037*** | |  | |  | |  | |  | |  | |  | |  |
|  |  |  | |  | | (0.012) | | (0.012) | | (0.010) | |  | |  | |  | |  | |  | |  | |  |
| Poor#Mid-reading country |  |  | |  | | -0.083*** | | -0.087*** | | -0.064*** | |  | |  | |  | |  | |  | |  | |  |
|  |  |  | |  | | (0.016) | | (0.017) | | (0.015) | |  | |  | |  | |  | |  | |  | |  |
| Poor#High-reading country |  |  | |  | | -0.045* | | -0.037 | | -0.084*** | |  | |  | |  | |  | |  | |  | |  |
|  |  |  | |  | | (0.022) | | (0.022) | | (0.021) | |  | |  | |  | |  | |  | |  | |  |
| **Location (base category: urban)** | -0.124*** | -0.120*** | | -0.114*** | | -0.185*** | | -0.185*** | | -0.168*** | | -0.075*** | | -0.081*** | | -0.045*** | | -0.115*** | | -0.112*** | | -0.106*** | |  |
|  | (0.009) | (0.009) | | (0.009) | | (0.008) | | (0.008) | | (0.008) | | (0.013) | | (0.013) | | (0.012) | | (0.009) | | (0.009) | | (0.009) | |  |
| Rural#Mid-reading country |  |  | |  | |  | |  | |  | | -0.029 | | -0.014 | | -0.047** | |  | |  | |  | |  |
|  |  |  | |  | |  | |  | |  | | (0.017) | | (0.017) | | (0.016) | |  | |  | |  | |  |
| Rural#High-reading country |  |  | |  | |  | |  | |  | | -0.117*** | | -0.104*** | | -0.164*** | |  | |  | |  | |  |
|  |  |  | |  | |  | |  | |  | | (0.018) | | (0.018) | | (0.018) | |  | |  | |  | |  |
| **Disabled (base category: non-disabled)** | -0.156*** | -0.164*** | | -0.140*** | | -0.169*** | | -0.178*** | | -0.153*** | | -0.158*** | | -0.167*** | | -0.143*** | | -0.131*** | | -0.142*** | | -0.111*** | |  |
|  | (0.015) | (0.015) | | (0.014) | | (0.015) | | (0.015) | | (0.014) | | (0.015) | | (0.015) | | (0.014) | | (0.021) | | (0.021) | | (0.019) | |  |
| Disabled#Mid-reading country |  |  | |  | |  | |  | |  | |  | |  | |  | | -0.03 | | -0.029 | | -0.026 | |  |
|  |  |  | |  | |  | |  | |  | |  | |  | |  | | (0.027) | | (0.028) | | (0.025) | |  |
| Disabled#High-reading country |  |  | |  | |  | |  | |  | |  | |  | |  | | -0.053 | | -0.041 | | -0.092* | |  |
|  |  |  | |  | |  | |  | |  | |  | |  | |  | | (0.042) | | (0.041) | | (0.039) | |  |
| **Age (Base category=10)** |  |  | |  | |  | |  | |  | |  | |  | |  | |  | |  | |  | |  |
| age11 | 0.063*** | 0.063*** | | 0.053*** | | 0.064*** | | 0.063*** | | 0.054*** | | 0.063*** | | 0.062*** | | 0.053*** | | 0.062*** | | 0.061*** | | 0.052*** | |  |
|  | (0.009) | (0.009) | | (0.009) | | (0.009) | | (0.009) | | (0.009) | | (0.009) | | (0.009) | | (0.009) | | (0.009) | | (0.009) | | (0.009) | |  |
| age12 | 0.112*** | 0.117*** | | 0.092*** | | 0.111*** | | 0.116*** | | 0.092*** | | 0.112*** | | 0.117*** | | 0.092*** | | 0.111*** | | 0.117*** | | 0.092*** | |  |
|  | (0.009) | (0.009) | | (0.008) | | (0.009) | | (0.009) | | (0.009) | | (0.009) | | (0.009) | | (0.008) | | (0.009) | | (0.009) | | (0.008) | |  |
| age13 | 0.163*** | 0.172*** | | 0.135*** | | 0.163*** | | 0.173*** | | 0.135*** | | 0.162*** | | 0.171*** | | 0.134*** | | 0.162*** | | 0.171*** | | 0.134*** | |  |
|  | (0.009) | (0.009) | | (0.008) | | (0.009) | | (0.009) | | (0.009) | | (0.009) | | (0.009) | | (0.009) | | (0.009) | | (0.009) | | (0.009) | |  |
| age14 | 0.207*** | 0.218*** | | 0.174*** | | 0.210*** | | 0.221*** | | 0.177*** | | 0.207*** | | 0.218*** | | 0.174*** | | 0.206*** | | 0.217*** | | 0.173*** | |  |
|  | (0.009) | (0.009) | | (0.009) | | (0.009) | | (0.009) | | (0.009) | | (0.009) | | (0.009) | | (0.009) | | (0.009) | | (0.009) | | (0.009) | |  |
| **Gender (Base category: Men)** | 0.038*** | 0.038*** | | 0.034*** | | 0.040*** | | 0.040*** | | 0.035*** | | 0.038*** | | 0.038*** | | 0.034*** | | 0.038*** | | 0.038*** | | 0.034*** | |  |
|  | (0.006) | (0.006) | | (0.006) | | (0.006) | | (0.006) | | (0.006) | | (0.006) | | (0.006) | | (0.006) | | (0.006) | | (0.006) | | (0.006) | |  |
| **Country** |  |  | |  | |  | |  | |  | |  | |  | |  | |  | |  | |  | |  |
|  | 0.314*** | 0.337*** | | 0.238*** | | 0.307*** | | 0.330*** | | 0.237*** | | 0.293*** | | 0.305*** | | 0.238*** | | 0.279*** | | 0.301*** | | 0.214*** | |  |
|  | (0.008) | (0.009) | | (0.008) | | (0.009) | | (0.009) | | (0.008) | | (0.014) | | (0.014) | | (0.013) | | 0.008 | | 0.008 | | 0.007 | |  |
|  | 0.478*** | 0.483*** | | 0.430*** | | 0.457*** | | 0.460*** | | 0.418*** | | 0.522*** | | 0.520*** | | 0.502*** | | 0.456*** | | 0.461*** | | 0.410*** | |  |
|  | (0.009) | (0.009) | | (0.010) | | (0.010) | | (0.010) | | (0.010) | | (0.013) | | (0.013) | | (0.014) | | 0.009 | | 0.009 | | 0.009 | |  |
| **Constant** | 0.009 | 0.017 | | -0.004 | | 0.113*** | | 0.126*** | | 0.094*** | | -0.040* | | -0.026 | | -0.058*** | | -0.019 | | -0.01 | | -0.025 | |  |
|  | (0.016) | (0.016) | | (0.016) | | (0.014) | | (0.015) | | (0.014) | | (0.017) | | (0.017) | | (0.016) | | 0.016 | | 0.016 | | 0.016 | |  |
| Sample size | 23572 | 23572 | | 23572 | | 23572 | | 23572 | | 23572 | | 23572 | | 23572 | | 23572 | | 23572 | | 23572 | | 23572 | |  |
| R2 | 0.208 | 0.215 | | 0.175 | | 0.192 | | 0.198 | | 0.163 | | 0.207 | | 0.214 | | 0.178 | | 0.206 | | 0.212 | | 0.175 | |  |

S2.5 IPW least squares regressions, interaction terms between various factors and country groups (continuous outcome variable)

|  | Family Schooling | Poverty Status | Urban Vs. Rural | Disability Status |
| --- | --- | --- | --- | --- |
| **Highest educational level in the household (base category=No school)** | | | | |
| Primary |  | 0.032*** | 0.025*** | 0.026*** |
|  |  | (0.007) | (0.007) | (0.007) |
| Junior secondary |  | 0.087*** | 0.059*** | 0.058*** |
|  |  | (0.008) | (0.008) | (0.008) |
| Senior secondary or higher |  | 0.106*** | 0.076*** | 0.075*** |
|  |  | (0.008) | (0.008) | (0.008) |
| No School | 0.056*** |  |  |  |
|  | (0.010) |  |  |  |
| No School#Mid-reading country | -0.163*** | |  |  |
|  | (0.013) |  |  |  |
| No School#High-reading country | -0.093*** | |  |  |
|  | (0.018) |  |  |  |
| **Wealth index (base category=Poorest)** | |  |  |  |
| Second quintile | 0.051*** |  | 0.050*** | 0.050*** |
|  | (0.008) |  | (0.008) | (0.008) |
| Middle | 0.089*** |  | 0.088*** | 0.089*** |
|  | (0.008) |  | (0.008) | (0.008) |
| Fourth quintile | 0.144*** |  | 0.142*** | 0.144*** |
|  | (0.008) |  | (0.009) | (0.009) |
| Richest | 0.241*** |  | 0.234*** | 0.236*** |
|  | (0.009) |  | (0.009) | (0.009) |
| Poor |  | -0.062*** | |  |
|  |  | (0.012) |  |  |
| Poor#Mid-reading country |  | -0.076*** | |  |
|  |  | (0.015) |  |  |
| Poor#High-reading country |  | -0.007 |  |  |
|  |  | (0.019) |  |  |
| **Location (base category: urban)** | -0.091*** | -0.147*** | -0.110*** | -0.083*** |
|  | (0.006) | (0.006) | (0.012) | (0.007) |
| Rural#Mid-reading country |  |  | 0.046*** |  |
|  |  |  | (0.014) |  |
| Rural#High-reading country |  |  | 0.020 |  |
|  |  |  | (0.015) |  |
| **Disabled (base category: non-disabled)** | -0.170*** | -0.182*** | -0.173*** | -0.175*** |
|  | (0.013) | (0.013) | (0.013) | (0.023) |
| Disabled#Mid-reading country |  |  |  | 0.004 |
|  |  |  |  | (0.028) |
| Disabled#High-reading country |  |  |  | -0.004 |
|  |  |  |  | (0.035) |
| **Age (Base category=10)** |  |  |  |  |
| age11 | 0.069*** | 0.069*** | 0.067*** | 0.067*** |
|  | (0.007) | (0.008) | (0.007) | (0.007) |
| age12 | 0.120*** | 0.119*** | 0.120*** | 0.120*** |
|  | (0.007) | (0.007) | (0.007) | (0.007) |
| age13 | 0.174*** | 0.175*** | 0.173*** | 0.173*** |
|  | (0.007) | (0.007) | (0.007) | (0.007) |
| age14 | 0.219*** | 0.221*** | 0.218*** | 0.218*** |
|  | (0.007) | (0.007) | (0.007) | (0.007) |
| **Gender (Base category: Men)** | 0.039*** | 0.041*** | 0.039*** | 0.039*** |
|  | (0.005) | (0.005) | (0.005) | (0.005) |
| **Country** |  |  |  |  |
|  | 0.341*** | 0.331*** | 0.275*** | 0.304*** |
|  | (0.008) | (0.008) | (0.011) | (0.007) |
|  | 0.462*** | 0.434*** | 0.427*** | 0.439*** |
|  | (0.008) | (0.008) | (0.011) | (0.008) |
| **Constant** | 0.125*** | 0.238*** | 0.118*** | 0.102*** |
|  | (0.013) | (0.012) | (0.014) | (0.013) |
| Sample size | 23572 | 23572 | 23572 | 23572 |
| R2 | 0.29 | 0.27 | 0.286 | 0.286 |

S2.6 IPW least squares regressions, interaction terms between disability status and social factors (outcome variable cutoff at 85%, 80%, and 90%)

| Cut point | 0.85 | 0.8 | 0.9 |
| --- | --- | --- | --- |
| **Disabled (base category: non-disabled)** | -0.249*** | -0.268*** | -0.249*** |
|  | (0.045) | (0.047) | (0.040) |
| **Location (base category: urban)** | -0.117*** | -0.114*** | -0.103*** |
|  | (0.009) | (0.009) | (0.009) |
|  |  |  |  |
| **Disabled # Location** |  |  |  |
| Disabled # Rural | 0.055 | 0.054 | 0.092** |
|  | (0.035) | (0.035) | (0.032) |
| **Wealth index (base category=Poorest)** | | |  |
| Middle | 0.081*** | 0.081*** | 0.076*** |
|  | (0.008) | (0.009) | (0.008) |
| Richest | 0.232*** | 0.238*** | 0.218*** |
|  | (0.012) | (0.012) | (0.012) |
|  |  |  |  |
| **Disabled # Wealth Index** | |  |  |
| Disabled#Middle | 0.000 | 0.02 | -0.003 |
|  | (0.034) | (0.035) | (0.031) |
| Disabled#Richest | 0.01 | 0.039 | 0.013 |
|  | (0.057) | (0.056) | (0.053) |
| Primary | 0.034*** | 0.032*** | 0.024** |
|  | (0.009) | (0.009) | (0.008) |
| Junior secondary | 0.109*** | 0.105*** | 0.092*** |
|  | (0.010) | (0.010) | (0.010) |
| Senior secondary or higher | 0.096*** | 0.095*** | 0.084*** |
|  | (0.011) | (0.011) | (0.011) |
|  |  |  |  |
| **Disabled # Highest Education level in the household** | | | |
| Disabled#1 | 0.094** | 0.086* | 0.058 |
|  | (0.036) | (0.037) | (0.031) |
| Disabled#2 | 0.049 | 0.056 | 0.041 |
|  | (0.039) | (0.041) | (0.034) |
| Disabled#3 | 0.073 | 0.048 | 0.098* |
|  | (0.050) | (0.051) | (0.046) |
| **Age (Base category=10)** | |  |  |
| age11 | 0.064*** | 0.063*** | 0.054*** |
|  | (0.009) | (0.009) | (0.009) |
| age12 | 0.111*** | 0.116*** | 0.092*** |
|  | (0.009) | (0.009) | (0.008) |
| age13 | 0.162*** | 0.172*** | 0.136*** |
|  | (0.009) | (0.009) | (0.008) |
| age14 | 0.206*** | 0.217*** | 0.174*** |
|  | (0.009) | (0.009) | (0.009) |
| **Gender (Base category: Boys)** | 0.036*** | 0.036*** | 0.032*** |
|  | (0.006) | (0.006) | (0.006) |
| **Country** |  |  |  |
| Chad | 0.065*** | 0.070*** | 0.068*** |
|  | (0.017) | (0.017) | (0.015) |
| DRCongo | 0.043** | 0.053*** | 0.037** |
|  | (0.014) | (0.014) | (0.013) |
| Ghana | 0.315*** | 0.332*** | 0.268*** |
|  | (0.015) | (0.015) | (0.014) |
| Lesotho | 0.466*** | 0.487*** | 0.360*** |
|  | (0.017) | (0.017) | (0.017) |
| Madagascar | 0.388*** | 0.431*** | 0.304*** |
|  | (0.016) | (0.016) | (0.015) |
| Malawi | 0.379*** | 0.413*** | 0.289*** |
|  | (0.014) | (0.015) | (0.014) |
| The Gambia | 0.221*** | 0.231*** | 0.187*** |
|  | (0.018) | (0.018) | (0.016) |
| Togo | 0.270*** | 0.280*** | 0.208*** |
|  | (0.017) | (0.017) | (0.015) |
| Tunisia | 0.698*** | 0.723*** | 0.636*** |
|  | (0.014) | (0.014) | (0.015) |
| Zimbabwe | 0.427*** | 0.424*** | 0.414*** |
|  | (0.015) | (0.015) | (0.015) |
| _cons | -0.097*** | -0.093*** | -0.100*** |
|  | (0.019) | (0.019) | (0.018) |
| Sample size | 23572 | 23572 | 23572 |
| R2 | 0.222 | 0.233 | 0.19 |

S2.7 IPW least squares regressions, interaction terms between disability status and social factors (continuous outcome variable)

|  | Cut point 0.85 | Continuous |  |  |
| --- | --- | --- | --- | --- |
| **Disabled (base category: non-disabled)** | -0.249*** | -0.263*** |  |  |
|  | (0.045) | 0.043 |  |  |
| **Location (base category: urban)** | -0.117*** | -0.093*** |  |  |
|  | (0.009) | 0.007 |  |  |
|  |  |  |  |  |
| **Disabled # Location** |  |  |  |  |
| Disabled # Rural | 0.055 | 0.008 |  |  |
|  | (0.035) | 0.029 |  |  |
| **Wealth index (base category=Poorest)** |  |  |  |  |
| Middle | 0.081*** | 0.085*** |  |  |
|  | (0.008) | 0.007 |  |  |
| Richest | 0.232*** | 0.213*** |  |  |
|  | (0.012) | 0.009 |  |  |
|  |  |  |  |  |
| **Disabled # Wealth Index** |  |  |  |  |
| Disabled#Middle | 0.000 | 0.065* |  |  |
|  | (0.034) | 0.032 |  |  |
| Disabled#Richest | 0.01 | 0.048 |  |  |
|  | (0.057) | 0.047 |  |  |
| **Highest educational level in the household (base category=No school)** | | | | |
| Primary | 0.034*** | 0.031*** |  |  |
|  | (0.009) | 0.008 |  |  |
| Junior secondary | 0.109*** | 0.096*** |  |  |
|  | (0.010) | 0.008 |  |  |
| Senior secondary or higher | 0.096*** | 0.093*** |  |  |
|  | (0.011) | 0.009 |  |  |
|  |  |  |  |  |
| **Disabled # Highest Education level in the household** | |  |  |  |
| Disabled#1 | 0.094** | 0.070* |  |  |
|  | (0.036) | 0.034 |  |  |
| Disabled#2 | 0.049 | 0.037 |  |  |
|  | (0.039) | 0.036 |  |  |
| Disabled#3 | 0.073 | 0.062 |  |  |
|  | (0.050) | 0.045 |  |  |
| **Age (Base category=10)** |  |  |  |  |
| age11 | 0.064*** | 0.070*** |  |  |
|  | (0.009) | 0.007 |  |  |
| age12 | 0.111*** | 0.120*** |  |  |
|  | (0.009) | 0.007 |  |  |
| age13 | 0.162*** | 0.175*** |  |  |
|  | (0.009) | 0.007 |  |  |
| age14 | 0.206*** | 0.219*** |  |  |
|  | (0.009) | 0.007 |  |  |
| **Gender (Base category: Men)** | 0.036*** | 0.036*** |  |  |
|  | (0.006) | 0.005 |  |  |
| **Country** |  |  |  |  |
| Chad | 0.065*** | 0.088*** |  |  |
|  | (0.017) | 0.016 |  |  |
| DRCongo | 0.043** | 0.071*** |  |  |
|  | (0.014) | 0.014 |  |  |
| Ghana | 0.315*** | 0.360*** |  |  |
|  | (0.015) | 0.013 |  |  |
| Lesotho | 0.466*** | 0.499*** |  |  |
|  | (0.017) | 0.015 |  |  |
| Madagascar | 0.388*** | 0.475*** |  |  |
|  | (0.016) | 0.014 |  |  |
| Malawi | 0.379*** | 0.436*** |  |  |
|  | (0.014) | 0.013 |  |  |
| The Gambia | 0.221*** | 0.271*** |  |  |
|  | (0.018) | 0.016 |  |  |
| Togo | 0.270*** | 0.280*** |  |  |
|  | (0.017) | 0.016 |  |  |
| Tunisia | 0.698*** | 0.643*** |  |  |
|  | (0.014) | 0.013 |  |  |
| Zimbabwe | 0.427*** | 0.475*** |  |  |
|  | (0.015) | 0.013 |  |  |
| **Constant** | -0.097*** | 0.007 |  |  |
|  | (0.019) | 0.016 |  |  |
| Sample size | 23572 | 23572 |  |  |
| R2 | 0.222 | 0.311 |  |  |
